# Effects of Silver Nanoparticles on the Red Microalga *Porphyridium purpureum* CNMN-AR-02, Cultivated on Two Nutrient Media

**DOI:** 10.3390/md22050208

**Published:** 2024-05-01

**Authors:** Ludmila Rudi, Liliana Cepoi, Tatiana Chiriac, Svetlana Djur, Ana Valuta, Vera Miscu

**Affiliations:** Institute of Microbiology and Biotechnology, Technical University of Moldova, 2028 Chisinau, Moldova; liliana.cepoi@imb.utm.md (L.C.); tatiana.chiriac@imb.utm.md (T.C.); svetlana.djur@imb.utm.md (S.D.); ana.valuta@imb.utm.md (A.V.); vera.miscu@imb.utm.md (V.M.)

**Keywords:** *Porphyridium purpureum*, silver nanoparticles, nutrient media, biomass, biochemical composition, antioxidant activity, malondialdehyde content

## Abstract

The purpose of this study was to examine the influence of 10 and 20 nm nanoparticles (AgNPs) on the growth and biochemical composition of microalga *Porphyridium purpureum* CNMN-AR-02 in two media which differ by the total amount of mineral salts (MM1 with 33.02 g/L and MM2 with 21.65 g/L). Spectrophotometric methods were used to estimate the amount of biomass and its biochemical composition. This study provides evidence of both stimulatory and inhibitory effects of AgNPs on different parameters depending on the concentration, size, and composition of the nutrient medium. In relation to the mineral medium, AgNPs exhibited various effects on the content of proteins (an increase up to 20.5% in MM2 and a decrease up to 36.8% in MM1), carbohydrates (a decrease up to 35.8% in MM1 and 39.6% in MM2), phycobiliproteins (an increase up to 15.7% in MM2 and 56.8% in MM1), lipids (an increase up to 197% in MM1 and no changes found in MM2), antioxidant activity (a decrease in both media). The composition of the cultivation medium has been revealed as one of the factors influencing the involvement of nanoparticles in the biosynthetic activity of microalgae.

## 1. Introduction

Interest in the use of microalga *Porphyridium purpureum* (formerly *Porphyridium cruentum*) (Rhodophyta) as a biotechnological object is steadily increasing, as it represents a valuable source of substances with pronounced biological effects, such as phycobiliproteins, carbohydrates, especially sulfated polysaccharides and lipids, which contain appreciable quantities of polyunsaturated fatty acids [1,2,3,4,5]. Being a marine species, microalga is cultivated under industrial conditions in media with high mineral salt content, and this involves both high production costs and negative environmental impacts due to the excessive discharge of saline wastewater. At the same time, it has been shown that under the conditions of a short biotechnological cycle, *Porphyridium purpureum* culture can provide the same technological advantages in terms of the quantity and quality of biomass, using various media with different mineral nutrient content, with a significantly lower mineral salt content [6,7].

Another aspect of improving *Porphyridium purpureum* cultivation technologies lies in the application of various procedures to stimulate biomass growth and direct biomass composition. In the context of applying stimulants to produce higher amounts of biomass with a valuable and predictable biochemical content, nanoparticles of various natures are increasingly being explored from this perspective [8,9]. For example, nanoparticles of Mg, Al, Zn, Cu, Pb, Ag, Fe, Fe_3_O_4_ are used as effective stimulants to enhance lipid synthesis in microalgae for biofuel production purposes [10,11,12]. However, this effect is more often a result of the stress caused by high concentrations of these nanoparticles. At the current stage, the greatest successes in the study of the interactions between nanoparticles and microalgae pertain to elucidating these entities’ toxicity mechanisms, with the metabolic pathways involved in the response reactions being highlighted [13]. At the same time, by applying low concentrations of nanoparticles, it is possible to accelerate the growth rate of microalgae, improve nutrient absorption, stimulate biomass production, and optimize the synthesis of bio-products of interest, such as proteins, carbohydrates, pigments, and lipids [8,14,15]. The remarkable bioavailability of nanoparticles supports the idea that they could represent more efficient sources of trace elements compared to their macroscopic forms, thereby contributing to improve vital functions [16].

Silver nanoparticles (AgNPs) are among the most actively used in various fields, such as the food industry, agriculture, textile industry, medical industry, and phycobiotechnologies, including for the valorization of *Porphyridium purpureum* biomass [8,11,17,18,19]. Thus, AgNPs are used as stimulants for biosynthetic activity, primarily lipids, the content of which can increase in microalgal biomass by 3–8 times [15,18]. Studies have shown the stimulating action of AgNPs on other biosynthetic processes such as carbohydrate production [8].

Modeling the cultivation conditions of microalgae growth in relation to the characteristics of nanoparticles, such as type, size, coating, and concentration, creates new opportunities to enhance the yield of biologically active substances [18,20]. In this context, the study of the influence characteristics of silver nanoparticles on microalga *Porphyridium purpureum* CNMN-AR-02 is of particular interest. The potential positive effects of silver nanoparticles can be amplified through the additional benefits offered by the possibility of applying a medium with a reduced content of mineral salts. Moreover, under such nutritional conditions, the effects of the nanoparticles can be significantly altered both in terms of magnitude and the direction of this effect.

Thus, the purpose of this study was to highlight the effects of two-dimensional nanoparticles—10 and 20 nm—applied in concentrations from 0.01 to 10.0 µM to the culture of *Porphyridium purpureum* CNMN-AR-02 on two mineral media characterized by different total salt content.

## 2. Results

### 2.1. Quantity of P. purpureum CNMN-AR-02 Biomass Produced under Culture Conditions with Various AgNPs Concentrations 

**Figure 1:**
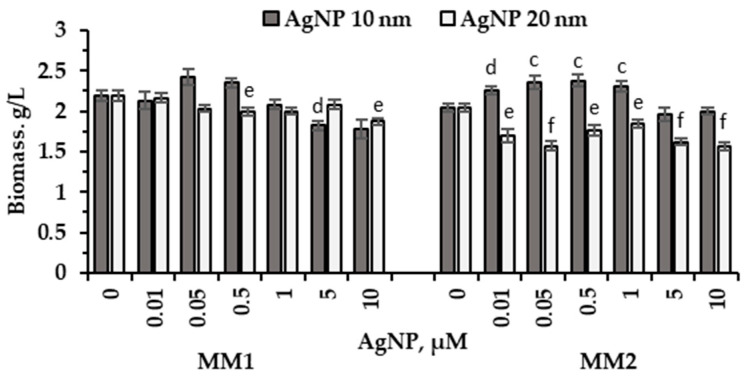
Biomass (g/L) of *P. purpureum* CNMN-AR-02 produced under conditions with different AgNPs concentrations (µM) in mineral media MM1 and MM2. 0: control sample. The letters “c” and “e” indicate a significant difference compared to the control (Student’s *t*-test, *p* < 0.01): “c” for 10 nm AgNPs; “e” for 20 nm AgNPs. The letters “d” and “f” indicate a highly significant difference compared to the control (Student’s *t*-test, *p* < 0.001): “d” for 10 nm AgNPs; “f” for 20 nm AgNPs. Error bars denote standard deviation (*n* = 3).

The amount of biomass produced during the cultivation of red microalgae *P. purpureum* CNMN-AR-02 in the presence of various AgNPs concentrations of 10 nm and 20 nm in size, supplemented into the nutrient media MM1 and MM2, is shown in Figure 1.

The growth of *P. purpureum* CNMN-AR-02 in MM1 mineral medium supplemented with 10 nm AgNPs at concentrations of 0.05 µM and 0.5 μM enhanced biomass production by 10.5% and 7.3%, respectively. In contrast, the supplementation of 5 µM and 10 µM to MM1 medium showed a significant decrease of 16.9% (*p* < 0.001) and 18.7%, respectively, in microalgae biomass. Regarding 20 nm AgNPs, their addition in 10 µM to MM1 medium led to a significant reduction in biomass quantity by 14.6%, compared to the control sample (*p* < 0.01). In the case of the MM2 medium, the use of 10 nm AgNPs in concentrations ranging from 0.01 to 1.0 µM led to a significant increase in biomass accumulation; values have been estimated from 10.8% (*p* < 0.001) to 16.7% (*p* < 0.01). In contrast to 10 nm particles, AgNPs of 20 nm in diameter significantly reduced the amount of microalgae biomass at all concentrations applied to MM2 medium; the values ranged from 9.8% (*p* < 0.01) to 23.5% (*p* < 0.001) below the control level. 

### 2.2. Composition of P. purpureum CNMN-AR- 02 Biomass Produced in the Presence of Various AgNPs Concentrations

**Figure 2:**
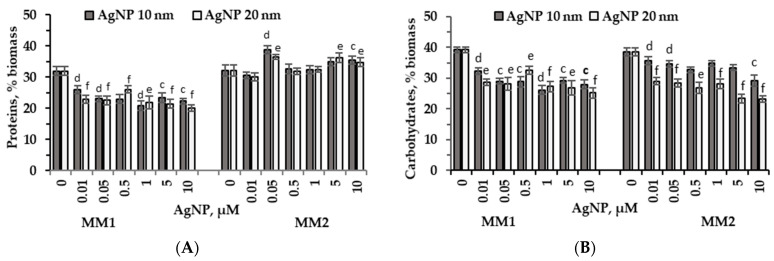
Protein, % biomass (**A**) and carbohydrate, % biomass (**B**) content in *P. purpureum* CNMN-AR-02 biomass during the cultivation under different AgNPs concentrations (µM) in mineral media MM1 and MM2. 0: control sample. The letters “c” and “e” indicate a significant difference compared to the control (Student’s *t*-test, *p* < 0.01): “c” for 10 nm AgNPs; “e” for 20 nm AgNPs. The letters “d” and “f” indicate a highly significant difference compared to the control (Student’s *t*-test, *p* < 0.001): “d” for 10 nm AgNPs; “f” for 20 nm AgNPs. Error bars denote standard deviation (*n* = 3).

The analysis of *P. purpureum* CNMN-AR-02 biomass composition established that 10 nm and 20 nm AgNPs, applied to MM1 cultivation medium, resulted in a significant decrease in protein content by 18.4% (*p* < 0.001) – 36.8% (*p* < 0.001) (Figure 2A). In the case of MM2 medium, the protein content increased compared to the control in *P. purpureum* CNMN-AR-02 biomass, with the maximum values of 20.5% (*p* < 0.01) and 13.4% (*p* < 0.01) determined for 10 nm and 20 nm AgNPs when applied at a concentration of 0.05 µM (Figure 2A).

Figure 2B presents changes in the carbohydrate content in *P. purpureum* CNMN-AR-02 biomass during cultivation in MM1 or MM2 mineral media and in the presence of silver nanoparticles. The cultivation of *P. purpureum* CNMN-AR-02 in MM1 medium with both types of AgNPs—10 nm and 20 nm—resulted in a significant reduction in the carbohydrate content of microalgal biomass. The decrease in carbohydrate values in the biomass collected at the end of the cultivation cycle was between 17.4% and 35.8% below the control level (*p* < 0.001). In the MM2 medium, the effect of silver nanoparticles was manifested by a moderate reduction compared to the control of the carbohydrate content in microalgal biomass, which was from 7.14% (*p* < 0.001) to 23.9% (*p* < 0.01) in the presence of 10 nm silver nanoparticles and from 24.8% to 39.6% in the presence of 20 nm AgNPs (*p* < 0.001). 

**Figure 3:**
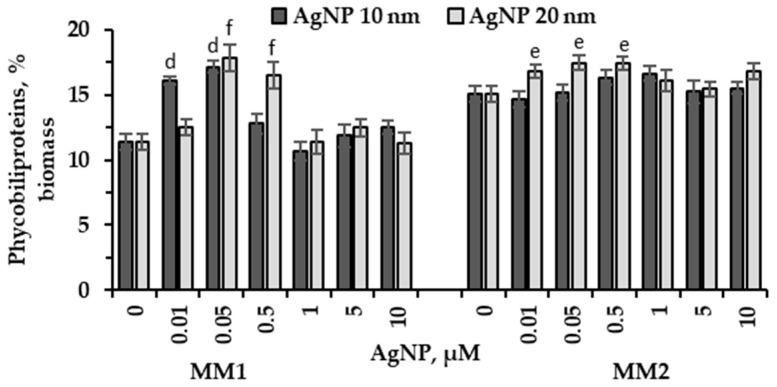
Phycobiliprotein content (% biomass) in *P. purpureum* CNMN-AR-02 biomass during cultivation under different AgNPs concentrations (µM) in mineral media MM1 and MM2. 0: control sample. The letter “e” indicates a significant difference compared to the control (Student’s *t*-test, *p* < 0.01) for 20 nm AgNPs. The letters “d” and “f” indicate a highly significant difference compared to the control (Student’s *t*-test, *p* < 0.001): “d” for 10 nm AgNPs; “f” for 20 nm AgNPs. Error bars denote standard deviation (*n* = 3).

The modification of phycobiliprotein content In biomass depending on the size and concentration of silver nanoparticles, during the cultivation of microalga *P. purpureum* CNMN-AR-02 on culture media MM1 and MM2, is presented in Figure 3.

In the MM1 medium, 10 nm AgNPs in concentrations of 0.01 µM and 0.05 µM induced significant changes in the phycobiliprotein content in microalgal biomass, which increased by 41.9%–50.7% compared to the control (*p* < 0.001). Concentrations ranging from 0.5 µM to 10 µM of AgNPs either resulted in a 12% increase in phycobiliprotein content or did not alter their content in the biomass. In the presence of 20 nm AgNPs in concentrations of 0.05 µM and 0.5 µM, the phycobiliprotein content increased by 56.8% and 45.3% compared to the control (*p* < 0.001). Concentrations between 1.0 µM and 10 µM of this type of nanoparticles did not change the accumulated levels of phycobiliproteins in *P. purpureum* CNMN-AR-02 biomass. AgNPs of 10 nm in size added to the MM2 medium had no significant effects on the phycobiliprotein content in the biomass of *P. purpureum* CNMN-AR-02. An increase of 10.2% was found for the nanoparticle concentration of 1.0 µM. Nanoparticles with a size of 20 nm induced an increase of 15.3%–15.7% in the content of pigments in the biomass of *P. purpureum* CNMN-AR-02 grown in the presence of concentrations of 0.05 µM and 0.5 µM (*p* < 0.001).

**Figure 4:**
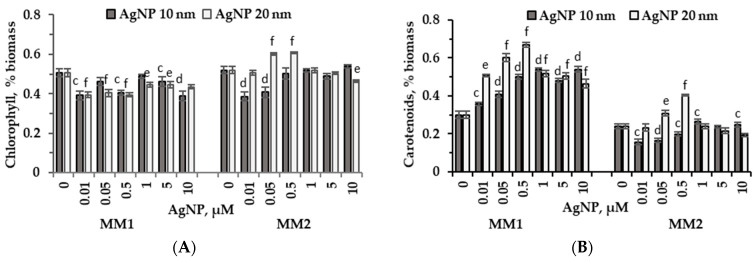
Chlorophyll, % biomass (**A**) and carotenoid, % biomass (**B**) content in *P. purpureum* CNMN-AR-02 biomass during cultivation under different AgNPs concentrations (µM) in mineral media MM1 and MM2. 0: control sample. The letters “c” and “e” indicate a significant difference compared to the control (Student’s *t*-test, *p* < 0.01): “c” for 10 nm AgNPs; “e” for 20 nm AgNPs. The letters “d” and “f” indicate a highly significant difference compared to the control (Student’s *t*-test, *p* < 0.001): “d” for 10 nm AgNPs; “f” for 20 nm AgNPs. Error bars denote standard deviation (*n* = 3).

The chlorophyll content determined in the microalgal biomass obtained under cultivation conditions of *P. purpureum* CNMN-AR-02 in the presence of 10 nm and 20 nm AgNPs in the MM1 medium differed from that determined in the biomass produced by *P. purpureum* CNMN-AR-02 in the MM2 medium (Figure 4A).

The silver nanoparticles introduced into the MM1 medium reduced the amount of chlorophyll in the biomass. The lowest chlorophyll content, 20.2%–23.2% below the control level, was recorded when MM1 nutrient medium was supplemented with 10 nm AgNPs at concentrations of 0.01 µM, 0.5 µM, and 10 µM (*p* < 0.01), as well as in the case of 0.01–0.5 µM concentrations of 20 nm AgNPs (*p* < 0.001). In MM2 medium, the concentrations of 0.01 µM and 0.05 µM of 10 nm AgNPs reduced chlorophyll content, which decreased by 25.7% and 21.7%, respectively (*p* < 0.001). The chlorophyll content in *P. purpureum* CNMN-AR-02 biomass grown in the presence of 20 nm AgNPs supplemented to MM2 nutrient medium in concentrations of 0.05 µM and 0.5 µM increased by 15.9% and 16.7%, respectively (*p* < 0.001) compared to the control. The concentration of 10 µM of this type of nanoparticle reduced the chlorophyll content by 10.8% (*p* < 0.01).

The carotenoid content in the microalgal culture underwent substantial changes (Figure 4B). In the MM1 medium, the *P. purpureum* CNMN-AR-02 culture showed a single response to all concentrations of applied silver nanoparticles—an increase in carotenoid production of 19.2% (*p* < 0.01)–80.5% (*p* < 0.01) in the case of 10 nm nanoparticles, and by 55%–124.4% in the case of 20 nm nanoparticles (*p* < 0.001). In the MM2 mineral medium, small concentrations of 10 nm AgNPs, in the range of 0.01–0.5 µM, reduced the carotenoid content in the algal biomass by 16.4%–34.4% (*p* < 0.01). Concentrations of 0.05 µM and 0.5 µM of 20 nm AgNPs increased carotenoid production by 29.4% (*p* < 0.01) and 69.7% (*p* < 0.001) compared to the control, respectively. 

**Figure 5:**
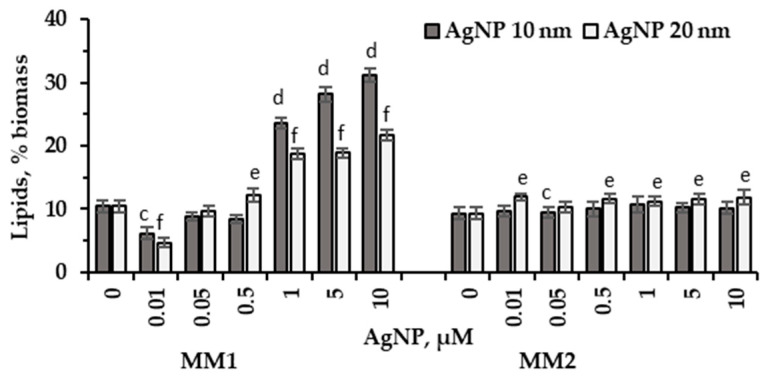
Lipid content (% biomass) in *P. purpureum* CNMN-AR-02 biomass during cultivation under different AgNPs concentrations (µM) in mineral media MM1 and MM2. 0: control sample. The letters “c” and “e” indicate a significant difference compared to the control (Student’s *t*-test, *p* < 0.01): “c” for 10 nm AgNPs; “e” for 20 nm AgNPs. The letters “d” and “f” indicate a highly significant difference compared to the control (Student’s *t*-test, *p* < 0.001): “d” for 10 nm AgNPs; “f” for 20 nm AgNPs. Error bars denote standard deviation (*n* = 3).

Silver nanoparticles of 10 nm in size, at concentrations of 1–10 µM, introduced into the MM1 cultivation medium stimulated lipid synthesis in *P. purpureum* CNMN-AR-02 culture. As a result, the lipid content increased by 2.25–2.97 times (*p* < 0.001) in the microalgal biomass compared to the control (Figure 5). The 20 nm nanoparticles in the same concentrations increased the lipid content by 78.6%–107.9% compared to the control sample (*p* < 0.001). Supplemented to the MM2 medium, 10 nm silver nanoparticles did not change the lipid content. AgNPs of 20 nm in size stimulated the synthesis of lipids, their content in microalgal biomass collected at the end of *P. purpureum* CNMN-AR-02 growth being 20%–27.5% higher compared to the control sample (*p* < 0.01).

### 2.3. MDA in P. purpureum CNMN-AR-02 Biomass Grown in the Presence of Silver Nanoparticles 

**Figure 6:**
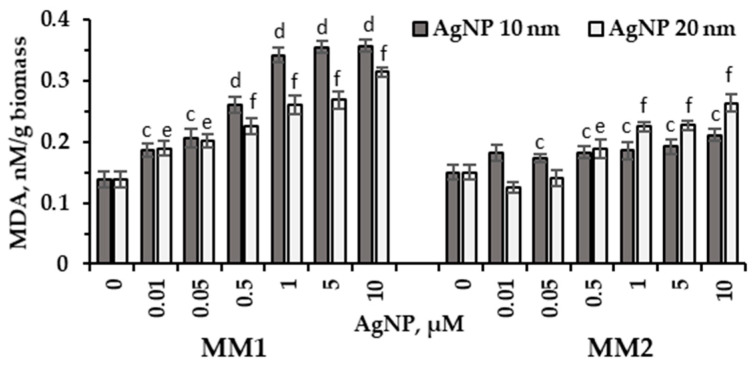
MDA content (nM/mg) in *P. purpureum* CNMN-AR-02 biomass obtained in the presence of AgNPs in mineral media MM1 and MM2. 0: control sample. The letters “c” and “e” indicate a significant difference compared to the control (Student’s *t*-test, *p* < 0.01): “c” for 10 nm AgNPs; “e” for 20 nm AgNPs. The letters “d” and “f” indicate a highly significant difference compared to the control (Student’s *t*-test, *p* < 0.001): “d” for 10 nm AgNPs; “f” for 20 nm AgNPs. Error bars denote standard deviation (*n* = 3).

In the conducted experiments, the level of lipid oxidative degradation products in microalgal biomass showed a significant increase in all experimental variants, as shown in Figure 6.

The values of MDA (malondialdehyde), a common indicator of oxidative stress and lipid damage, were determined in *P. purpureum* CNMN-AR-02 biomass cultivated in two different media, MM1 and MM2. In MM1 medium, significant increases in MDA values were observed depending on the concentration of silver nanoparticles (AgNPs) applied. Concentrations of AgNPs ranging between 0.01 and 0.5 µM resulted in MDA content increases of 34.8% (*p* < 0.01)–89% (*p* < 0.001) for 10 nm AgNPs and of 36.9% (*p* < 0.01)–63.7% (*p* < 0.001) for 20 nm AgNPs compared to the control. Higher concentrations of 1–10 µM of 10 nm and 20 nm AgNPs induced significant increases in MDA values in microalgal biomass, by 2.48–2.58 times and of 89%–127.5%, respectively (*p* < 0.001). On the other hand, in MM2 medium, the application of silver nanoparticles led to moderate increases in MDA values. In this case, all concentrations of 10 nm AgNPs increased MDA content by 15.3%–40.7% (*p* < 0.001), while high concentrations of 20 nm AgNPs resulted in increases of 26%–75.3% (*p* < 0.001). Low concentrations of 20 nm AgNPs did not significantly affect the MDA content in microalgal biomass grown in MM2 culture medium. 

### 2.4. Antioxidant Activity P. purpureum CNMN-AR-02 Grown in the Presence of Silver Nanoparticles 

**Figure 7:**
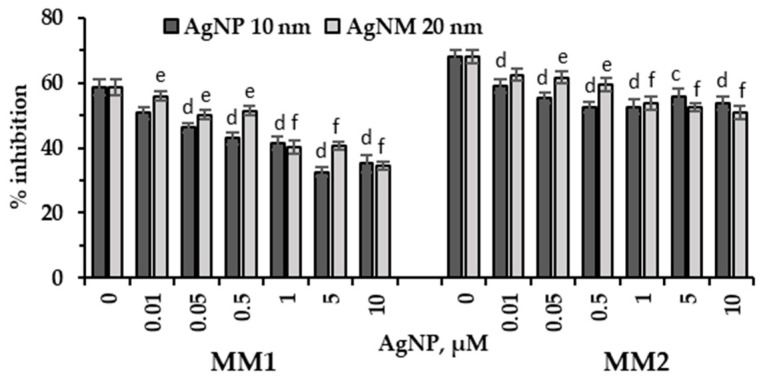
Antioxidant activity (% inhibition) of the aqueous extracts derived from *P. purpureum* CNMN-AR-02 biomass grown under different AgNPs concentrations (µM) in mineral media MM1 and MM2. 0: control sample. The letters “c” and “e” indicate a significant difference compared to the control (Student’s *t*-test, *p* < 0.01): “c” for 10 nm AgNPs; “e” for 20 nm AgNPs. The letters “d” and “f” indicate a highly significant difference compared to the control (Student’s *t*-test, *p* < 0.001): “d” for 10 nm AgNPs; “f” for 20 nm AgNPs. Error bars denote standard deviation (*n* = 3).

The addition of silver nanoparticles to both cultivation media of *P. purpureum* CNMN-AR-02 led to a different manner of reducing the antioxidant activity of the aqueous extracts derived from microalgae biomass (Figure 7).

Silver nanoparticles with a size of 10 nm applied into MM1 medium resulted in a significant reduction in antioxidant activity of aqueous extracts obtained from *P. purpureum* CNMN-AR-02 biomass. The level of reduction in antioxidant activity varied between 13.4% (*p* < 0.01) and 44.4% (*p* < 0.001) below that of the control sample, relative to the increase in nanoparticle concentration. Silver nanoparticles of 20 nm caused a lesser degree of reduction in antioxidant activity, only by 14.3% and 12.3% (*p* < 0.01) for concentrations of 0.01 and 0.5 µM, and a more pronounced decrease of 31.5–41.5% (*p* < 0.001) when concentrations of 1.0–10 µM were applied. In the aqueous extracts obtained from *P. purpureum* CNMN-AR-02 biomass grown in the presence of silver nanoparticles in MM2 medium, antioxidant activity decreased more moderately, by 13.4–23% (*p* < 0.001) (10 nm AgNP) and by 8.4–25.5% (*p* < 0.001) (20 nm AgNP), compared to the control.

## 3. Discussion

In this research, two mineral media were used to cultivate red microalga *P. purpureum*. These nutrient media were different in terms of their mineral composition: mineral medium MM1 comprised 33.02 g/L salts and mineral medium MM2 comprised 21.65 g/L salts. Under standard conditions of cultivation on two media, certain differences were observed both in the amount of biomass and in its composition at the end of the vital cycle. Thus, in the control sample in MM1 medium, biomass accumulation was higher by 7.35% compared to MM2 medium (Figure 1). The content of proteins, lipids, carbohydrates and chlorophyll in *P. purpureum* biomass obtained in the control sample remained unaltered regardless of the two media formulations. However, a significant difference was observed in the abundance of phycobiliproteins in the case of using MM2 medium—it was 32.8% higher compared to MM1 medium (Figure 3), and the content of carotenoids was, on the contrary, 20.4% higher in MM1 medium (Figure 4B). Differences in the composition of *P. purpureum* CNMN-AR-02 biomass depending on the salinity level in the nutrient medium were also found by other researchers. For instance, *P. purpureum* biomass grown in a nutrient medium with 32 g/L NaCl contained 77% more carbohydrates compared to biomass produced in the medium with a higher salinity level of 50 g/L NaCl and 2.3 times more proteins than in microalgal biomass grown in a medium with 18 g/L NaCl [21]. A 2.97 times increase of lipid content in *P. purpureum* CNMN-AR-02 biomass was recorded in the case of MM1 medium with a chloride content of 15.26 g/L in combination with AgNPs. The lipid content in microalgae biomass grown in MM2 mineral medium in the presence of AgNPs and a chloride content of 7.82 g/L did not change.

Under these conditions, it can be assumed that the responses of *P. purpureum* CNMN-AR-02 to various factors will also vary depending on the nutrient medium used for its cultivation. In this research, silver nanoparticles of 10 and 20 nm in size were investigated as influencing factors. It is widely known that nanoparticles are easily absorbed by living systems due to their small size, and their effects depend on several factors, including their sizes and concentration [22]. 

The analysis of the size-dependent action of silver nanoparticles showed a stimulating effect on microalgae biomass production in the case of 10 nm AgNPs (Figure 1). Thus, the application of low concentrations of 0.01, 0.05, 0.5, and 1.0 μM only to MM2 nutrient medium induced an increase in biomass up to 17% compared to the control sample. Concentrations of 5.0 and 10.0 μM AgNPs of 10 nm in the MM1 medium caused a reduction in the amount of biomass, whereas this effect was absent in MM2. Several concentrations (0.05 μM and 0.5 μM) have been identified as stimulators in both culture media, with the MM2 medium being more favorable. Nanoparticles of 20 nm in size exhibited completely different effects, leading to a significant decrease in the amount of biomass of *P. purpureum* CNMN-AR-02 in MM2 medium, while this effect was absent or poorly expressed in MM1.

The influence of the nutrient medium formulation on microalgae response in terms of biomass composition was very obvious. Thus, AgNPs of both sizes and in all concentrations introduced into MM1 medium caused a pronounced decrease in the content of proteins in biomass. At the same time, the use of MM2 medium showed no changes in the protein content or a significant increase in this parameter compared to the control (Figure 2A). 

Regarding the size-dependent effect of NPs on the amount of carbohydrates in microalgae biomass, both 10 nm and 20 nm AgNPs were found to exhibit similar effects, with the size in particular determining the intensity of the effect (Figure 2B). Thus, the addition of 10 nm AgNPs to the MM2 medium led to a decrease in the carbohydrate content of *P. purpureum* CNMN-AR-02 biomass by 7–23.9%, and AgNPs with a size of 20 nm by 24.8–39.6% compared to the control. In MM1 medium, AgNPs of both sizes caused significant reductions in carbohydrate content compared to the control sample, but no differences were observed depending on the size and concentration of NPs.

The content of pigments in microalgae biomass usually undergoes very significant changes under the action of various environmental factors. This was explained by the fact that pigments, in addition to the basic function of capturing light energy in order to use it for photosynthesis, also perform protective functions: antioxidant protection and photoprotection, thereby they are synthesized and consumed in cells even according to the level of stress [23]. In our study, the MM1 medium differed in that the resulting biomass had a low content of phycobiliproteins compared to the MM2 medium (Figure 3). Silver nanoparticles in concentrations up to 0.5 μM applied to MM1 medium depending on their size induced a significant increase in the amount of phycobiliproteins in algal biomass by 41.8–50.6%. Regarding MM2 medium, only 20 nm AgNPs in concentrations of 0.01, 0.05, and 0.5 μM promoted an increase in phycobiliprotein content. The mineral composition of MM2 medium provided a higher content of phycobiliproteins in the absence of nanoparticles, and their presence led to a moderate synthesis of these pigments.

Under cultivation conditions of *P. purpureum* CNMN-AR-02 in MM1 medium, a reduction in chlorophyll level up to 23% was found in biomass, as well as an increase in the carotene content for all tested concentrations (Figure 4A,B). Adding 10 nm AgNPs to the MM2 medium resulted in a moderate decrease in the content of these two pigments at concentrations up to 0.5 μM, while other concentrations did not alter the chlorophyll content and moderately enhanced carotenoid production in microalgal biomass. At the same time, 20 nm AgNPs in concentrations of 0.05 and 0.5 µM stimulated the synthesis of chlorophyll by 17% and carotenoids by 29.4% and 69.7%, without altering these parameters upon microalgae exposure to other tested concentrations. Some studies have shown changes in the photosynthetic system of microalgae under the action of nanoparticles. Notably, in the culture of microalga *Nannochloropsis oculate* (Eustigmatophyceae), concentrations of 5–50 mg/L AgNPs with an average size of 54.8 nm led to a reduction in chlorophyll content and an increase in carotenoid production [24]. However, other studies have reported an increase in chlorophyll level upon contact of microalgae with nanoparticles. Thus, the growth of *Tetradesmus obliquus* (formerly *Scenedesmus obliquus)* (Chlorophyta) in the presence of 5–10 mg/L Fe_2_O_3_ NPs enhanced chlorophyll content in biomass by 10–17% [25]. The carotenoid content in the biomass of green microalga *Dunaliella salina* (Chlorophyta) increased by 1.48 times in the presence of MoS_2_ NPs at a concentration of 50 µg/L. This effect was achieved due to a synergistic combination with the light intensity factor [26].

Microalgae cultures undergo significant changes in the lipid content of biomass under the action of various stress factors, in particular nanoparticles. For example, concentrations of 5 and 20 μg/L of 6–10 nm AgNPs increased the lipid content of *Scenedesmus* sp. up to 81%, and in the case of microalgae *Talassiosira* sp. (Mediophyceae), lipidogenesis was stimulated by the concentrations of 100, 200 mg/L of these nanoparticles [14]. It was noticed that exposure to iron oxide (III) nanoparticles with a size of 30 nm resulted in increased lipid content up to 39.6% culture of *Tetradesmus obliquus* [24]. Fe_2_O_3_ NPs with a size of 50 nm enhanced biomass and lipid accumulation in *Chlorella* sp. (Chlorophyta) cultures by 33.75% and 15.29%, respectively [27].

In this study, the effect of silver nanoparticles on the lipid content of *P. purpureum* CNMN-AR-02 was quite pronounced in MM1 medium and moderate in MM2 medium (Figure 5). Nanoparticles of both sizes stimulate lipidogenesis in *P. purpureum* CNMN-AR-02 grown in MM1 medium starting from the concentration of 1.0 μM. In this culture medium, 10 nm AgNPs have been found to induce a significantly higher increase in lipid content. Silver nanoparticles with a size of 20 nm in MM2 medium exerted a moderate stimulating effect on lipidogenesis. This highlights that nutrient limitation in the culture medium of microalgae *P. purpureum* CNMN-AR-02 allowed for a reduction of the toxic effects of AgNPs. The effect of silver nanoparticles to enhance lipid synthesis turned out to be possible under conditions of microalgae cultivation in MM1 mineral medium. This enriched mineral medium improved biosynthetic activity, and silver nanoparticles redirected this process toward lipid synthesis.

The level of oxidative stress in the culture of *P. purpureum* CNMN-AR-02 was expressed by an increase in the content of malondialdehyde (MDA) (Figure 6). In the case of MM1 medium, a significant increase in MDA content was detected in all experimental variants, while in MM2 medium the increase in MDA occurred as an overall effect, starting with the concentration of 0.5 μM silver nanoparticles, but was rather moderate compared to MM1 medium.

We mention that in the biomass of *P. purpureum* CNMN-AR-02 grown in MM1 medium, silver nanoparticles in concentrations of 1–10 μM caused a reduction of up to 36.8% in protein content and 35.8% in carbohydrate content, while lipid content significantly increased (Figure 2A,B and Figure 5).

Under these conditions, high MDA values were strongly positively correlated with lipid content (r^2^ = 0.945 for 10 nm AgNPs and r^2^ = 0.962 for 20 nm AgNPs). The involvement of different concentrations of NPs in redirecting biosynthetic activity of microalgae has been revealed in other studies. For example, a concentration of 20 mg/L Fe_2_O_3_ nanoparticles significantly stimulated the productivity of microalgae *Chlorella* sp. (Chlorophyta) UJ-3, while the maximum lipid content was achieved at high nanoparticle concentrations of 100 mg/L [20].

When applying AgNPs in all concentrations in MM2 medium, no decreases in the amount of proteins in the biomass of *P. purpureum* CNMN-AR-02 were recorded, and the amount of lipids under the same conditions was up to 20.7% (Figure 2A and Figure 5). In this case, a weak correlation was established between elevated MDA values and lipid content (r^2^ = 0.468 for 10 nm AgNPs and r^2^ = 0.338 for 20 nm AgNPs). AgNPs acted differently depending on their concentration and the composition of the culture medium of *P. purpureum* CNMN-AR-02. In the case of MM2 medium, the effect of nanoparticles on lipidogenesis was largely determined by their presence, and not by their concentration within the tested concentrations.

Antioxidant activity can be considered an indicator of nanoparticle toxicity [28]. Regarding the control samples, the antioxidant activity was significantly lower compared to that achieved in MM2 medium (Figure 7). The results showed a reduction in antioxidant test values for all concentrations of AgNPs added to both culture media, indicating an obvious toxic effect of silver nanoparticles on the culture of *P. purpureum* CNMN-AR-02. The most drastic reduction in antioxidant activity by 50% occurred when microalga was grown in MM1 medium. 

The relationship between the action of nanoparticles and the level of nutrient supply of microalgae has been reported in several studies. Some authors noted that nanoparticles in the cultivation medium can affect the availability of macro- and microelements, as well as the rapid assimilation of nutrients. Thus, aluminum nanoparticles enhanced the growth of microalgae *Chlorella* sp., which accumulated 19% more biomass over a 4-day interval [29]. The impact of Zn oxide nanoparticles with a size of 100 nm on microalga *Chlorococcum* sp. (Chlorophyta) was demonstrated depending on the nitrate content in the cultivation medium and the duration of contact. The growth process was affected by the concentration of nanoparticles and the initial nitrate content in the cultivation medium. Extreme quantities, meaning both high and low levels of nitrates, have been shown to increase the toxicity of nanoparticles [30]. Growing microalga *P. purpureum* CNMN-AR-02 in MM1 medium with a nitrate content of 0.17 g/L and the addition of 10 nm AgNPs at a concentration of 10 µM led to a reduction in the amount of biomass of 18.7%. In the case of MM2 medium containing 0.8 g/L nitrates and in the experimental variant with 20 nm AgNPs, a decrease in the amount of biomass of 23.5% was determined.

In the case of halophilic algae, the salinity of the medium was a factor influencing the biological effects of nanoparticles. For example, concentrations of 0.5 and 1 mg/L AgNPs of 12.65 nm identically reduced the biomass production of microalga *Dunaliella salina* under salinity conditions of 35 and 70 g/L. A salinity of 140 g/L has favored biomass production in the presence of the respective concentrations of AgNPs [31].

## 4. Materials and Methods

### 4.1. The Strain of Red Microalga Porphyridium purpureum, Mineral Media, and Cultivation Conditions

Research was conducted on *Porphyridium purpureum* (formerly *Porphyridium cruentum*) strain CNMN-AR-02 stored in the National Collection of Non-Pathogenic Microorganisms, Technical University of Moldova, Institute of Microbiology and Biotechnology. Cultivation was carried out under photoautotrophic conditions. Two mineral media with different quantities of macro- and micronutrients were used. The macroelement contents in the cultivation media is presented in Table 1.

To 1000 mL formulated medium MM1, 1 mL of micronutrient solution consisting of 2.86 mg/L H_3_BO_3_; 1.81 mg/L MnCI_2_·4H_2_O; 0.08 mg/L CuSO_4_·5H_2_O; 0.015 mg/L MoO_3_, and 0.5 mL FeEDTA solution was added. For the MM2 mineral medium, the 1 mL micronutrient solution consisted of 2.7 mg/L FeCl_3_·6H_2_O; 0.02 mg/L ZnSO_4_·7H_2_O; 0.05 mg/L CuSO_4_·5H_2_O; 0.3 mg/L MnSO_4_·5H_2_O; 0.6 mg/L H_3_BO_3_; 0.02 mg/L MoO_3_; 0.05 mg/L NaVO_3_.

Experiments were carried out in 100 mL Erlenmeyer flasks containing 50 mL growth microalgae culture. The amount of inoculum was 0.45–0.50 g/L dry biomass. The cultivation of microalgae under laboratory conditions was performed by maintaining the following parameters: temperature of 25–28 °C, optimal pH in the culture medium of 6.8–7.2, and continuous illumination of 56 µmol photons m^−2^ s^−1^. The cultivation cycle lasted 14 days. 

### 4.2. Silver Nanoparticles

For experimental purposes, citrate-stabilized silver nanoparticles with sizes of 10 nm (Lot # MKCK8345, product of USA) and 20 nm (Lot #MKCM2276, Product USA) (SIGMA-ALDRICH CHEMIE GmbH, Steinheim, Germany) were used. The specifications of the nanoparticles are OD 1 and PDI < 0.2. The nanoparticle sizes were validated by TEM. The accuracy of nanoparticle size is ensured by an error of ± 0.2 nm. 

### 4.3. Collection and Standardization of Microalgal Biomass

At the end of the cultivation cycle, the microalgal biomass from each sample was separated from culture medium by centrifugation and subjected to demineralization (removal of excess salts from the cell surface) by washing with 2% ammonium acetate solution. The biomass samples were standardized to a concentration of 10 mg/mL. To ensure the availability of the cellular content for biochemical tests, the biomass samples were treated through a repeated freeze/thaw procedure. 

### 4.4. Determination of Biomass Amount

The amount of *Porphyridium purpureum* biomass was determined by measuring the absorbance of the microalgal suspension at 545 nm with quantitative recalculation in g/L based on the calibration curve.

### 4.5. Determination of the Biochemical Composition of Microalgal Biomass

#### 4.5.1. Protein Content

The protein content in biomass was determined based on the Lowry method. To extract the protein, 0.9 mL of 0.1 N sodium hydroxide was added to 10 mg of biomass. The extraction process took place at room temperature for 30 min. To 0.1 mL of protein extract, 1.6 mL of 2% sodium carbonate in 0.1 N sodium hydroxide, 0.4 mL of 0.5% copper sulfate in 1% sodium acetate, and 0.2 mL of Folin-Ciocalteu reagent were added. After the incubation time of 30 min, absorbance was recorded at 750 nm. Protein content was calculated based on the calibration curve for bovine serum albumin.

#### 4.5.2. Carbohydrates Content

For estimating carbohydrates, 2.5 mL of 0.5% anthrone solution in 66% sulfuric acid was added to 0.25 mL of 10 mg/mL biomass suspension. The samples were incubated in a water bath at 100 °C for 30 min. After cooling, absorbance was recorded at 620 nm. Quantitative calculation was made based on the calibration curve for glucose.

#### 4.5.3. Phycobiliproteins Content

Phycobiliproteins were determined in the aqueous extract. Samples subjected to repeated pretreatment (8 times) by freezing (−20 °C)/thawing (approximately 25 °C) were centrifuged. Pigments were quantified by recording absorbance at 650 nm (allophycocyanin), 620 nm (phycocyanin), and 565 nm (phycoerythrin). Quantitative calculation was made based on equations using specific absorption coefficients [32].

#### 4.5.4. Chlorophyll and Carotenoid Content

Chlorophyll and carotenoid content was established in ethanolic extracts from microalgal biomass. To 10 mg biomass sample, 1 mL of 96% ethyl alcohol was added. The extraction was performed by shaking for 180 min. The supernatant was separated and absorbance was recorded at 664.1 nm, 648.6 nm, and 470 nm. The calculation was made based on equations using specific absorption coefficients [33].

#### 4.5.5. Lipid Content

The total lipid quantity was determined spectrophotometrically using the phospho-vanillin reagent. For lipid extraction from the microalgal biomass, 1.0 mL of chloroform/ethanol mixture (0.9*v*/0.1*v*) was added to the 10 mg sample. The extraction process was carried out by shaking at room temperature for 120 min. The obtained lipid extract was dried. To the lipid sample, 1.0 mL of concentrated sulfuric acid was added. Hydrolysis was carried out at 90 °C for 20 min. A 0.1 mL sample of the hydrolysate was mixed with 3.0 mL of phospho-vanillin reagent (1.2 mg vanillin in 1.0 mL 68% phosphoric acid). After the incubation period in darkness, the absorbance of the samples was recorded at a wavelength of 520 nm. The lipid content was calculated based on the calibration curve for pure oleic acid.

### 4.6. Determination of the Content of Lipid Peroxidation Products—MDA Test

For determining the content of malondialdehyde, to 10 mg of biomass, 3.0 mL of 0.76% thiobarbituric acid in 20% trichloroacetic acid was added. The obtained mixture was incubated in a water bath at 95 °C for 40 min. The samples were cooled and centrifuged. The absorbance of the supernatant was recorded at 532 nm and 600 nm. The quantitative calculation of MDA was performed using the molar extinction coefficient.

### 4.7. Evaluation of Antioxidant Activity

The antioxidant activity was assessed based on the reduction reaction of ABTS radical (2,2′-azino-bis (3-ethylbenzothiazoline-6-sulfonic acid)) identified for aqueous extracts derived from microalgal biomass pretreated by repeated freezing/thawing cycles. For each sample, 0.3 mL of supernatant was mixed with 2.7 mL of ABTS radical reagent (working solution with an absorbance of 0.700 ± 0.02 at 734 nm). The ABTS radical was generated by oxidation of 7 mM ABTS solution with 2.45 mM (*v*/*v*) potassium persulfate. The radical generation reaction lasted 12–16 h in darkness. The absorbance of the reagent mixture was determined at the end of the 6 min antioxidant reaction period at 734 nm. The values of antioxidant activity were expressed as % inhibition of the ABTS radical cation.

### 4.8. Statistical Analysis

The experiments were performed in triplicate. The experimental results were expressed as the average ± SD. To reveal differences between the control and experimental conditions, Student’s *t*-test was applied (applicable when comparing two means). A *p*-value of ≤0.01 was considered statistically significant. All statistical analyses were performed using Microsoft Excel software 2019, MSO 16.0.10406.20006.

## 5. Conclusions

The effects of citrate-stabilized 10 and 20 nm Ag nanoparticles were analyzed in relation to two nutrient media used for the cultivation of microalgae *P. purpureum* CNMN-AR-02, while the MM2 medium had limited nutrient content. As a common response to the action of AgNPs of the culture of *P. purpureum* CNMN-AR-02, grown on both nutrient media, we mentioned the reduction of carbohydrate content and antioxidant test values. At the same time, a decrease in the harmful effects of silver nanoparticles was observed when microalga was grown in MM2 medium. Silver nanoparticles added to MM1 mineral medium in concentrations of 1–10 μM have proven to be inductors of lipidogenesis in the microalgae strain, which can be applied for biotechnological purposes to produce biomass with high lipid content. Thus, the integration of nanoparticles into microalgal applied biotechnologies opens up prospects for improving the efficiency and sustainability of valuable compound production for various purposes, and one of the factors regulating the involvement of nanoparticles in the biosynthetic activity of microalgae is the composition of the cultivation medium. Given that the microalga *Porphyridium purpureum* is characterized by remarkable properties, high productivity, and versatile responses to external stimuli, studies in the biotechnology field of this subject hold practical significance and fundamental interest. 

## Figures and Tables

**Table 1:** The macroelement contents.

Macroelements Composition, g/L		
	MM1	MM2
KCl	16.04	7.5
NaCl	12.52	7.0
KNO_3_	1.24	-
NaNO_3_	-	5.0
MgSO_4_·7H_2_O	2.5	1.8
CaCl2	0.118	-
Ca(NO_3_)_2_·4H_2_O	-	0.15
K_2_HPO_4_·3H_2_O	0.5	0.2
KI	0.05	0.05
KBr	0.05	0.05
**Total salts**	**33.018**	**21.65**
**Cl**	**15.19**	**7.82**
**N**	**0.17**	**0.8**
**P**	**0.16**	**0.06**

## Data Availability

The original contributions presented in the study are included in the article, further inquiries can be directed to the corresponding author.

## References

[1] Liberti D., Imbimbo P., Giustino E., D’Elia L., Silva M., Barreira L., Monti D.M. (2023). Shedding Light on the Hidden Benefit of *Porphyridium cruentum* Culture. Antioxidants.

[2] Tsvetanova F., Yankov D. (2022). Bioactive Compounds from Red Microalgae with Therapeutic and Nutritional Value. Microorganisms.

[3] Yin H.C., Sui J.K., Han T.L., Liu T.Z., Wang H. (2022). Integration bioprocess of B-phycoerythrin and exopolysaccharides production from photosynthetic microalga *Porphyridium cruentum*. Front. Mar. Sci..

[4] Casas-Arrojo V., Decara J., de los Ángeles Arrojo-Agudo M., Pérez-Manríquez C., Abdala-Díaz R.T. (2021). Immunomodulatory, antioxidant activity and cytotoxic effect of sulfated polysaccharides from *Porphyridium cruentum*. (S.F. Gray) Nägeli. Biomolecules.

[5] Kiran B.R., Venkata Mohan S. (2021). Microalgal Cell Biofactory—Therapeutic, Nutraceutical and Functional Food Applications. Plants.

[6] Mehariya S., Goswami R.K., Karthikeysan O.P., Verma P. (2021). Microalgae for high-value products: A way towards green nutraceutical and pharmaceutical compounds. Chemosphere.

[7] Li T., Xu J., Wu H., Jiang P., Chen Z., Xiang W. (2019). Growth and Biochemical Composition of *Porphyridium purpureum* SCS-02 under Different Nitrogen Concentrations. Mar. Drugs.

[8] Cepoi L., Rudi L., Chiriac T., Valuta A., Zinicovscaia I., Miscu V., Rudic V. (2022). Silver Nanoparticles as Stimulators in Biotechnology of Porphyridium cruentum. Proceedings of the 5th International Conference on Nanotechnologies and Biomedical Engineering. Proceedings of ICNBME-2021.

[9] Huang Y., Gao M., Wang W., Liu Z., Qian W., Chun Chen C., Zhu X., Cai Z. (2022). Effects of manufactured nanomaterials on algae: Implications and applications. Front. Environ. Sci. Eng..

[10] Hasnain M., Munir N., Abideen Z., Dias D.A., Aslam F., Mancinelli R. (2023). Applying silver nanoparticles to enhance metabolite accumulation and biodiesel production in new algal resources. Agriculture.

[11] Rana A., Parmar A.S. (2023). Re-exploring silver nanoparticles and its potential applications. Nanotechnol. Environ. Eng..

[12] Dev Sarkar R., Singh H.B., Chandra Kalita M. (2021). Enhanced lipid accumulation in microalgae through nanoparticle-mediated approach, for biodiesel production: A mini-review. Heliyon.

[13] Cao M., Wang F., Zhou B., Chen H., Yuan R., Ma S., Geng H., Xing B. (2020). Mechanisms of photoinduced toxicity of AgNPs to the microalgae *Chlorella pyrenoidosa* in the presence of hematite nanoparticles: Insights from transcriptomics, metabolomics and the photochemical index. Environ. Sci. Nano.

[14] Yap J.K., Sankaran R., Chew K.W., Halimatul Munawaroh H.S., Ho S.H., Rajesh Banu J., Show P.L. (2021). Advancement of green technologies: A comprehensive review on the potential application of microalgae biomass. Chemosphere.

[15] Pham L. (2019). Effect of Silver Nanoparticles on Tropical Freshwater and Marine Microalgae. J. Chem..

[16] Komazec B., Cvjetko P., Balen B., Letofsky-Papst I., Lyons D.M., Peharec Štefanić P. (2023). The Occurrence of Oxidative Stress Induced by Silver Nanoparticles in *Chlorella vulgaris* Depends on the Surface-Stabilizing Agent. Nanomaterials.

[17] Nie P., Yu Z., Xu H. (2023). Synthesis, applications, toxicity and toxicity mechanisms of silver nanoparticles: A review. Ecotoxicol. Environ. Saf..

[18] Rudi L., Cepoi L., Chiriac T., Miscu V., Valuta A., Djur S. (2023). Effects of citrate-stabilized gold and silver nanoparticles on some safety parameters of *Porphyridium cruentum* biomass. Front. Bioeng. Biotechnol..

[19] Dawadi S., Katuwal S., Gupta A., Lamichhane U., Thapa R., Jaisi S., Lamichhane G., Bhattarai D., Parajuli N. (2021). Current Research on Silver Nanoparticles: Synthesis, Characterization, and Applications. J. Nanomater..

[20] Wang F., Liu T., Guan W., Xu L., Huo S., Ma A., Zhuang G., Terry N. (2021). Development of a Strategy for Enhancing the Biomass Growth and Lipid Accumulation of *Chlorella* sp. UJ-3 Using Magnetic Fe_3_O_4_ Nanoparticles. Nanomaterials.

[21] Decamp A., Martineau E., Grizeau D., Pruvost J., Gonçalves O. (2023). Effects of the salinity on the biosynthesis of the polysaccharides of the marine microalgae *Porphyridium cruentum*. Algal Res..

[22] Vargas-Estrada L., Torres-Arellano S., Longoria A., Arias D.M., Okoye P.U., Sebastian P.J. (2020). Role of nanoparticles on microalgal cultivation: A review. Fuel.

[23] Sun X.M., Ren L.J., Zhao Q.Y., Ji X.Y., Huang H. (2018). Microalgae for the production of lipid and carotenoids: A review with focus on stress regulation and adaptation. Biotechnol. Biofuels.

[24] Fazelian N., Movafeghi A., Yousefzadi M., Rahimzadeh M., Zarei M. (2020). Impact of silver nanoparticles on the growth, fatty acid profile, and antioxidative response of *Nannochloropsis oculata*. Acta Physiol. Plant..

[25] He M., Yan Y., Pei F., Wu M., Gebreluel T., Zou S., Wang C. (2021). Improvement on lipid production by *Scenedesmus obliquus* triggered by low dose exposure to nanoparticles. Sci. Rep..

[26] Luo S.W., Alimujiang A., Cui J., Chen T.T., Balamurugan S., Zheng J.W., Wang X., Yang W.D., Li H.Y. (2021). Molybdenum disulfide nanoparticles concurrently stimulated biomass and β-carotene accumulation in *Dunaliella salina*. Bioresour. Technol..

[27] Rana M.S., Bhushan S., Sudhakar D.R., Prajapati S.K. (2020). Effect of iron oxide nanoparticles on growth and biofuel potential of *Chlorella* spp. Algal Res..

[28] Yuan X., Gao X., Liu C., Liang W., Xue H., Li Z., Jin H. (2023). Application of Nanomaterials in the Production of Biomolecules in Microalgae: A Review. Mar. Drugs.

[29] Dey N., Vickram S., Thanigaivel S., Manikandan S., Subbaiya R., Karmegam N., Kim W., Govarthanan M. (2023). Aftermath of nanomaterials on lipid profile of microalgae as a radical fuel supplement—A review. Fuel.

[30] Tzanakis N., Aravantinou A.F., Manariotis I.D. (2023). Short-Term Toxicity of ZnO Nanoparticles on Microalgae at Different Initial Nutrient Concentrations. Sustainability.

[31] Johari S.A., Sarkheil M., Tayemeh M.B., Veisi S. (2018). Influence of salinity on the toxicity of silver nanoparticles (AgNPs) and silver nitrate (AgNO_3_) in halophilic microalgae, *Dunaliella salina*. Chemosphere.

[32] Huang Z., Zhong C., Dai J., Li S., Zheng M., He Y., Wang M., Chen B. (2021). Simultaneous enhancement on renewable bioactive compounds from *Porphyridium cruentum* via a novel two-stage cultivation. Algal Res..

[33] Lichtenthaler H.K. (1987). Chlorophylls and Carotenoids: Pigments of Photosynthetic Biomembranes. Methods Enzymol..

